# GATA1 activates HSD17B6 to improve efficiency of cisplatin in lung adenocarcinoma via DNA damage

**DOI:** 10.1186/s41021-024-00321-9

**Published:** 2024-12-18

**Authors:** Xingxing Shao, Hailang Hou, Huijie Chen, Wan Xia, Xinpu Geng, Jindao Wang

**Affiliations:** Pulmonary and Critical Care Medicine, Huaian Hospital of Huaian City, Huaian Cancer Hospital, No. 19 Shanyang Avenue, Huai’an District, Huai’an, 223200 China

**Keywords:** GATA1, HSD17B6, DNA damage, Lung adenocarcinoma, Cisplatin resistance

## Abstract

**Background:**

Lung adenocarcinoma (LUAD) is the most common histological type of non-small cell lung cancer (NSCLC). Platinum-based chemotherapy, such as cisplatin chemotherapy, is the cornerstone of treatment for LUAD patients. Nevertheless, cisplatin resistance remains the key obstacle to LUAD treatment, for its mechanism has not been fully elucidated.

**Methods:**

HSD17B6 mRNA expression data were accessed from TCGA-LUAD database and differential expression analysis was performed. Enrichment analysis of HSD17B6 was conducted by GSEA, and its upstream transcription factors were predicted by hTFtarget. mRNA and protein expression levels of HSD17B6 and GATA1 were assayed by qRT-PCR and WB, and the binding relationship between them was verified by chromatin immunoprecipitation assay and dual luciferase reporter assay. Cell viability and IC_50_ value of cisplatin-treated cells were measured by cell counting kit-8 assay. Cell cycle was assayed by flow cytometry. DNA damage level and DNA damage marker γ-H2AX expression were assayed by comet assay and western blot, respectively.

**Results:**

HSD17B6 was lowly expressed in LUAD tissues and cells and mainly enriched in homologous recombination and mismatch repair pathways. As cell function experiments revealed, overexpression of HSD17B suppressed malignant phenotypes and cisplatin resistance in LUAD cells through DNA damage. Bioinformatics analysis revealed that GATA1 is the upstream regulator of HSD17B6, which was markedly reduced in LUAD tissues and cells. ChIP and dual luciferase reporter assays ascertained the binding of GATA1 to HSD17B6. Knockdown of GATA1 attenuated the effect of overexpression of HSD17B6 on LUAD cell behaviors and cisplatin resistance.

**Conclusion:**

Transcription factor GATA1 could activate HSD17B6 to inhibit cisplatin resistance in LUAD through DNA damage, suggesting that GATA1/HSD17B6 axis may be a potential therapeutic target for chemotherapy resistance in LUAD patients.

**Supplementary Information:**

The online version contains supplementary material available at 10.1186/s41021-024-00321-9.

## Introduction

With the highest incidence and mortality among all cancers, lung cancer emerges as one of the most prevalent cancers globally [1]. According to the latest global cancer statistics, the 5-year overall survival of patients with lung cancer is only 10% to 20% in most countries [2]. Non-small cell lung cancer (NSCLC) covers about 85% of lung cancer cases [3]. The most prevalent histological type of NSCLC is lung adenocarcinoma (LUAD), whose most effective treatment is pneumonectomy. Whereas, patients’ survival rate and quality of life after pneumonectomy are not satisfactory [4]. Adjuvant platinum-based therapy is beneficial for prolonged survival and improved quality of life of lung cancer patients [5]. Therefore, platinum-containing drugs are often utilized as first-line chemotherapeutic drugs for adjuvant treatment of LUAD, such as cisplatin [6]. Cisplatin functions mainly by triggering DNA damage [7]. Nevertheless, resistance of cancer cells to cisplatin is a major impediment to successful chemotherapy [8]. Several potential mechanisms of chemoresistance in cancer cells such as DNA repair, reduced mismatch repair, apoptosis defects, and anti-apoptotic factors have been put forwards [9]. Wang et al. [10] reported that USP22 induced cisplatin resistance in LUAD by modulating DNA damage repair mediated by γH2AX and apoptosis mediated by Ku70/Bax. Huang et al. [11] reported that MALAT1 inhibits DNA damage and makes NSCLC cells resistant to cisplatin through BRCA1. In summary, the clinical research on drug mechanism resistance has gradually deepened. How to transform these research results into treatment methods is an urgent problem to be solved. Hence, this study will further clarify the mechanism of LUAD cisplatin resistance and proffer a novel target for the curing of malignant tumor chemotherapy resistance.

The HSD17B6 gene encodes a protein called hydroxysteroid 17-β dehydrogenase 6, which can convert 3α-androstanediol into dihydrotestosterone (DHT), and its abnormal level is linked with the progression of multiple tumors [12]. HSD17B6 has been suggested to hamper tumor progression. As Tian *et* al. [12] found, HSD17B6 can hamper the proliferation, migration, invasion, epithelial-mesenchymal transition and radiation resistance of LUAD cells. Lv et al. [13] discovered that HSD17B6 can inhibit the malignant progression of hepatocellular carcinoma cells. Nevertheless, effect of HSD17B6 on LUAD cisplatin resistance remains an open issue. Hence, we dived into the molecular mechanism of HSD17B6 resistance to cisplatin in LUAD, suggesting that HSD17B6 may be an underlying therapeutic target for LUAD chemotherapy resistance.

We probed into the role of GATA1 and HSD17B6 in cisplatin resistance of LUAD cells and proffered a new mechanism of cisplatin resistance in LUAD cells. Cell experiments confirmed that HSD17B6 had low expression in LUAD, while overexpression of HSD17B6 could inhibit LUAD cisplatin resistance through DNA damage. Further studies have ascertained that HSD17B6 has an upstream regulatory molecule GATA1, which could activate HSD17B6 to inhibit LUAD cisplatin resistance through DNA damage. In conclusion, our study enriched the role of GATA1/HSD17B6 regulatory axis in LUAD and bred new insights into solving clinical cisplatin resistance in LUAD in the future.

## Materials and methods

### Bioinformatics analyses

mRNA expression data were acquired from TCGA-LUAD database (https://portal.gdc.cancer.gov/) (normal: 59, tumor: 535). Differentially expressed mRNAs were obtained by edgeR package (4.2.0). The hTFtarget (http://bioinfo.life.hust.edu.cn/hTFtarget#!/) was used to predict the upstream potential transcription factors, and the JASPAR (http://jaspar.genereg.net/) database was utilized to forecast the binding sites between target gene and transcription factor to identify the transcription factor. The target gene expression data was downloaded from the TCGA database, samples were categorized into target gene high expression group and target gene low expression group using the median value. Gene set enrichment analysis (GSEA) (https://www.gsea-msigdb.org/) was utilized to conduct functional enrichment analysis on target gene.

### Cell culture

Human LUAD cell lines (H1299, H1975 and A549) and human bronchial epithelial cells (BEAS-2B) were bought from ATCC (USA) in March 2023. Cancer cells were grown in RPMI-1640 medium (Gibco, USA) containing 10% fetal bovine serum, whereas BEAS-2B cells were grown in serum-free LHC-9 medium (Gibco, USA). All cells were grown in a humidified incubator (5% CO_2_, 37 ℃), with the medium replaced every 3 days, and after 3 passages, the cells were used for subsequent experiments.

### Cell transfection

Small interfering RNA targeting GATA1 (si-GATA1) (F: GCACAGAGCAUGGCCUCCAGATT, R: UCUGGAGGCCAUGCUCUGUGCTT), oe-GATA1 (Table 1), oe-HSD17B6 (Table 2), and corresponding negative control si-NC, oe-NC were purchased from Ribobio (China). The plasmid and small interfering RNA to be transfected were mixed with transfection reagent Lipofectamine 2000 (Thermo Fisher, USA) according to the instructions of the kit and added to LUAD cells in order to silence or overexpress GATA1 or HSD17B6 in the cells. After 24 h, transfected cells were used for the next experiment.

**Table 1:** The sequence of oe-GATA1

oe-GATA1: ATGGA GTTCCCTGGC CTGGGGTCCC TGGGGACCTC AGAGCCCCTC CCCCAGTTTG TGGATCCTGC TCTGGTGTCC TCCACACCAG AATCAGGGGT TTTCTTCCCC TCTGGGCCTG AGGGCTTGGA TGCAGCAGCT TCCTCCACTG CCCCGAGCAC AGCCACCGCT GCAGCTGCGG CACTGGCCTA CTACAGGGAC GCTGAGGCCT ACAGACACTC CCCAGTCTTT CAGGTGTACC CATTGCTCAA CTGTATGGAG GGGATCCCAG GGGGCTCACC ATATGCCGGC TGGGCCTACG GCAAGACGGG GCTCTACCCT GCCTCAACTG TGTGTCCCAC CCGCGAGGAC TCTCCTCCCC AGGCCGTGGA AGATCTGGAT GGAAAAGGCA GCACCAGCTT CCTGGAGACT TTGAAGACAG AGCGGCTGAG CCCAGACCTC CTGACCCTGG GACCTGCACT GCCTTCATCA CTCCCTGTCC CCAATAGTGC TTATGGGGGC CCTGACTTTT CCAGTACCTT CTTTTCTCCC ACCGGGAGCC CCCTCAATTC AGCAGCCTAT TCCTCTCCCA AGCTTCGTGG AACTCTCCCC CTGCCTCCCT GTGAGGCCAG GGAGTGTGTG AACTGCGGAG CAACAGCCAC TCCACTGTGG CGGAGGGACA GGACAGGCCA CTACCTATGC AACGCCTGCG GCCTCTATCA CAAGATGAAT GGGCAGAACA GGCCCCTCAT CCGGCCCAAG AAGCGCCTGA TTGTCAGTAA ACGGGCAGGT ACTCAGTGCA CCAACTGCCA GACGACCACC ACGACACTGT GGCGGAGAAA TGCCAGTGGG GATCCCGTGT GCAATGCCTG CGGCCTCTAC TACAAGCTAC ACCAGGTGAA CCGGCCACTG ACCATGCGGA AGGATGGTAT TCAGACTCGA AACCGCAAGG CATCTGGAAA AGGGAAAAAG AAACGGGGCT CCAGTCTGGG AGGCACAGGA GCAGCCGAAG GACCAGCTGG TGGCTTTATG GTGGTGGCTG GGGGCAGCGG TAGCGGGAAT TGTGGGGAGG TGGCTTCAGG CCTGACACTG GGCCCCCCAG GTACTGCCCA TCTCTACCAA GGCCTGGGCC CTGTGGTGCT GTCAGGGCCT GTTAGCCACC TCATGCCTTT CCCTGGACCC CTACTGGGCT CACCCACGGG CTCCTTCCCC ACAGGCCCCA TGCCCCCCAC CACCAGCACT ACTGTGGTGG CTCCGCTCAG CTCATGA

**Table 2:** The sequence of oe-HSD17B6

oe-HSD17B6: ATGTGGCTCT ACCTGGCGGC CTTCGTGGGC CTGTACTACC TTCTGCACTG GTACCGGGAG AGGCAGGTGG TGAGCCACCT CCAAGACAAG TATGTCTTTA TCACGGGCTG TGACTCGGGC TTTGGGAACC TGCTGGCCAG ACAGCTGGAT GCACGAGGCT TGAGAGTGCT GGCTGCGTGT CTGACGGAGA AGGGGGCCGA GCAGCTGAGG GGCCAGACGT CTGACAGGCT GGAGACGGTG ACCCTGGATG TTACCAAGAT GGAGAGCATC GCTGCAGCTA CTCAGTGGGT GAAGGAGCAT GTGGGGGACA GAGGACTCTG GGGACTGGTG AACAATGCAG GCATTCTTAC ACCAATTACC TTATGTGAGT GGCTGAACAC TGAGGACTCT ATGAATATGC TCAAAGTGAA CCTCATTGGT GTGATCCAGG TGACCTTGAG CATGCTTCCT TTGGTGAGGA GAGCACGGGG AAGAATTGTC AATGTCTCCA GCATTCTGGG AAGAGTTGCT TTCTTTGTAG GAGGCTACTG TGTCTCCAAG TATGGAGTGG AAGCCTTTTC AGATATTCTG AGGCGTGAGA TTCAACATTT TGGGGTGAAA ATCAGCATAG TTGAACCTGG CTACTTCAGA ACGGGAATGA CAAACATGAC ACAGTCCTTA GAGCGAATGA AGCAAAGTTG GAAAGAAGCC CCCAAGCATA TTAAGGAGAC CTATGGACAG CAGTATTTTG ATGCCCTTTA CAATATCATG AAGGAAGGGC TGTTGAATTG TAGCACAAAC CTGAACCTGG TCACTGACTG CATGGAACAT GCTCTGACAT CGGTGCATCC GCGAACTCGA TATTCAGCTG GCTGGGATGC TAAATTTTTC TTCATCCCTC TATCTTATTT ACCTACATCA CTGGCAGACT ACATTTTGAC TAGATCTTGG CCCAAACCAG CCCAGGCAGT CTAA	

### qRT-PCR

Trizol (Beyotime, China) was utilized to extract total RNA. cDNA was synthesized utilizing PrimeScript RT Master Mix (TaKaRa, Japan), qRT-PCR was conducted on ABI QuantStudio 5 (Thermo Fisher, USA) system utilizing Power SYBR Green kit (TaKaRa, Japan), taking GAPDH as a standardized endogenous control. All outcomes were calculated by 2^−ΔΔCt^. Primer sequences are listed in Table 3.

**Table 3:** The qRT-PCR primers sequences

Gene	Primer sequence (5’ → 3’)
HSD17B6	F: CTCCAGCATTCTGGGAAGAG
	R: AAGAAGCCCCCAAGCATATT
GATA1	F: GGAGACTTTGAAGACAGAG
	R: GGAGAGGAATAGGCTGCTG
GAPDH	F: GGAGCGAGATCCCTCCAAAAT
	R: GGCTGTTGTCATACTTCTCATGG

### Western blot (WB)

WB was performed according to previous method [10] and repeated three times. The primary antibodies were rabbit anti-human HSD17B6 (1: 1000, #14669 T, cell signaling technology, USA), rabbit anti-human GATA1 (1:1000, ab133274, Abcam, UK), rabbit anti-human γ-H2AX (1:1000, ab229914, Abcam, UK), GAPDH (1:10000, ab181602, Abcam, UK), and the secondary antibody was goat anti-rabbit IgG H&L (HRP) (1:2000, ab6721, Abcam, UK).

### Cell counting kit-8 (CCK-8) assay

Cell viability was evaluated using CCK-8. In brief, the cells were put in 96-well plates at an initial density of 2 × 10^3^ cells/well, the original medium was discarded after 0, 1, 2, 3, and 4 days of culture. Each well was added with 90 μL of fresh serum-free medium and 10 μL of CCK-8 reagent (Beyotime, China) at 37 ℃ for 1 h. Absorbance was measured at a wavelength of 450 nm by a microplate reader (Promega, USA), and three biological experiment replicates were conducted for each set of experiments.

The sensitivity of A549 to cisplatin was determined by CCK-8. In brief, the cells were put into 96-well plates (1 × 10^4^) and cultured for 24 h. After treatment with cisplatin at different concentrations (0, 2, 5, 10, 15, 20 and 25 μg/mL), each well was added with 10 μL CCK-8 reagent (Beyotime, China) and cultured at 37 ℃ for 2 h. Optical density value at 450 nm wavelength was detected and the IC_50_ value was calculated [14].

### Flow cytometry (FCM)

Cell cycle was assayed by FCM. Cells were collected for experiment, treated with trypsin, rinsed with PBS, and then fixed with cold ethanol. Cells were stained with propidium iodide (Sigma, USA) for 15 min, and the proportion of cells in each period was assayed by FCM (Beckman Coulter, USA) [15].

### Comet experiment

Comet assay was conducted by a single cell gel electrophoresis kit. In short, the transfected cells were mounted on a comet slide by utilizing low melting-point agarose, lysed for 2 h at 4 ℃, and then subjected to 25 V electrophoresis in alkaline electrophoresis buffer (1 mmol/L EDTA, 300 mmol/L NaOH) for 30 min. Finally, the gels were neutralized with Tris–HCl buffer (0.4 mmol/L, PH = 7.5, 3 times, 10 min) and stained with PI. At last, cells were photographed by an Olympus BX51 fluorescence microscope (Olympus, Japan) and the comet tails were analyzed by CASP software [16].

### Chromatin immunoprecipitation (ChIP)

ChIP was conducted utilizing Active Motif Kit (USA). Cells (2 × 10^7^) were fixed in 1% formaldehyde for 10 min at room temperature, rinsed with PBS and lysed using the lysis buffer in the kit. After ultrasonic treatment, the protein-DNA complex was incubated with antibody-coupled protein G beads at 4 ℃ overnight. The antibodies used were anti-GATA1 (ab181544, abcam, UK) and anti-IgG antibodies (ab172730, abcam, UK). The next day, DNA was eluted with 1% SDS/0.1 mol/L NaHCO3, cross-linked reversely at 65 ℃, purified by phenol/chloroform extraction and ethanol precipitation, and subjected to qPCR [17]. Primer sequences are listed in Table 4.

**Table 4:** ChIP-qRT-PCR primers sequences

Primer Sets	Primer sequence (5’ → 3’)
Primer pair	F: GGGGCTGAGGAGCATACAAG
	R: CCCACTCCAACCTCTGCATT

### Dual-luciferase assay

pGL3 vector (Promega, USA) was used to analyze HSD17B6 promoter. HSD17B6-promoter-WT (**···**CACTTATTATCTTTTTT**···**) and HSD17B6-promoter-MUT (**···**CACTTAAAATCTATTTT**···**) plasmids were co-transfected with oe-GATA1 and oe-NC, respectively, utilizing lipofectamine 2000 (Thermo Fisher, USA). After 48 h, luciferase activity was measured using a dual-luciferase reporter system (yuanye Bio-Technology, China) [18].

### Statistical analysis

Statistical analysis was conducted by GraphPad 8.0. Differences between the two groups were measured by Student’s t-test. One-way analysis of variance was utilized to compare three or more groups. Each experiment was repeated three times. Data were expressed as mean ± standard deviation (SD). *P* < 0.05 indicated significant difference.

## Results

### HSD17B6 is lowly expressed in LUAD tissues and cells

**Fig. 1:**
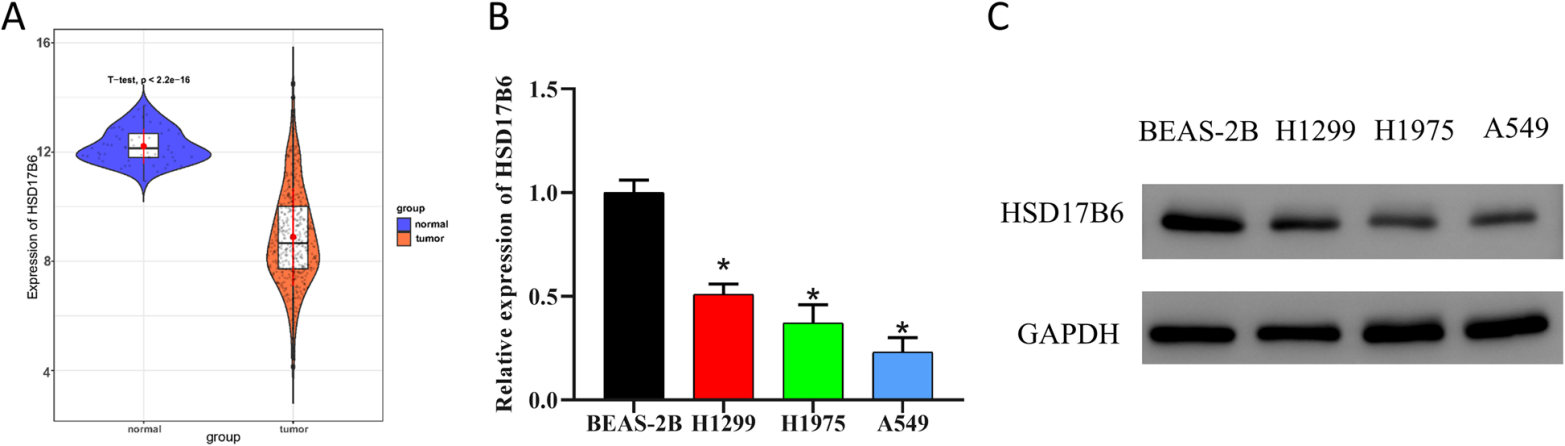
HSD17B6 shows decreased expression in LUAD tissues and cells. **A** TCGA analysis of HSD17B6 expression in normal and cancer tissues; **B**, **C** HSD17B6 expression in human LUAD cell lines and human bronchial epithelial cells by qRT-PCR and WB. * means *P* < 0.05

HSD17B6 has been ascertained to be down-regulated in liver cancer and NSCLC [13, 19]. We probed into the expression of HSD17B6 in LUAD, finding that HSD17B6 was lowly expressed in LUAD tissues by t-test (Fig. 1A). Expression of HSD17B6 in human LUAD cell lines H1299, H1975 and A549, and human bronchial epithelial cell BEAS-2B was detected by qRT-PCR and WB. The results indicating that HSD17B6 expression in LUAD cells was prominently down-regulated (Fig. 1B, C). As HSD17B6 expressed the lowest in A549 cells, this cell line was selected for subsequent experiments. These results revealed the decreased expression of HSD17B in LUAD tissues and cells.

### HSD17B6 inhibits LUAD cisplatin resistance through DNA damage

**Fig. 2:**
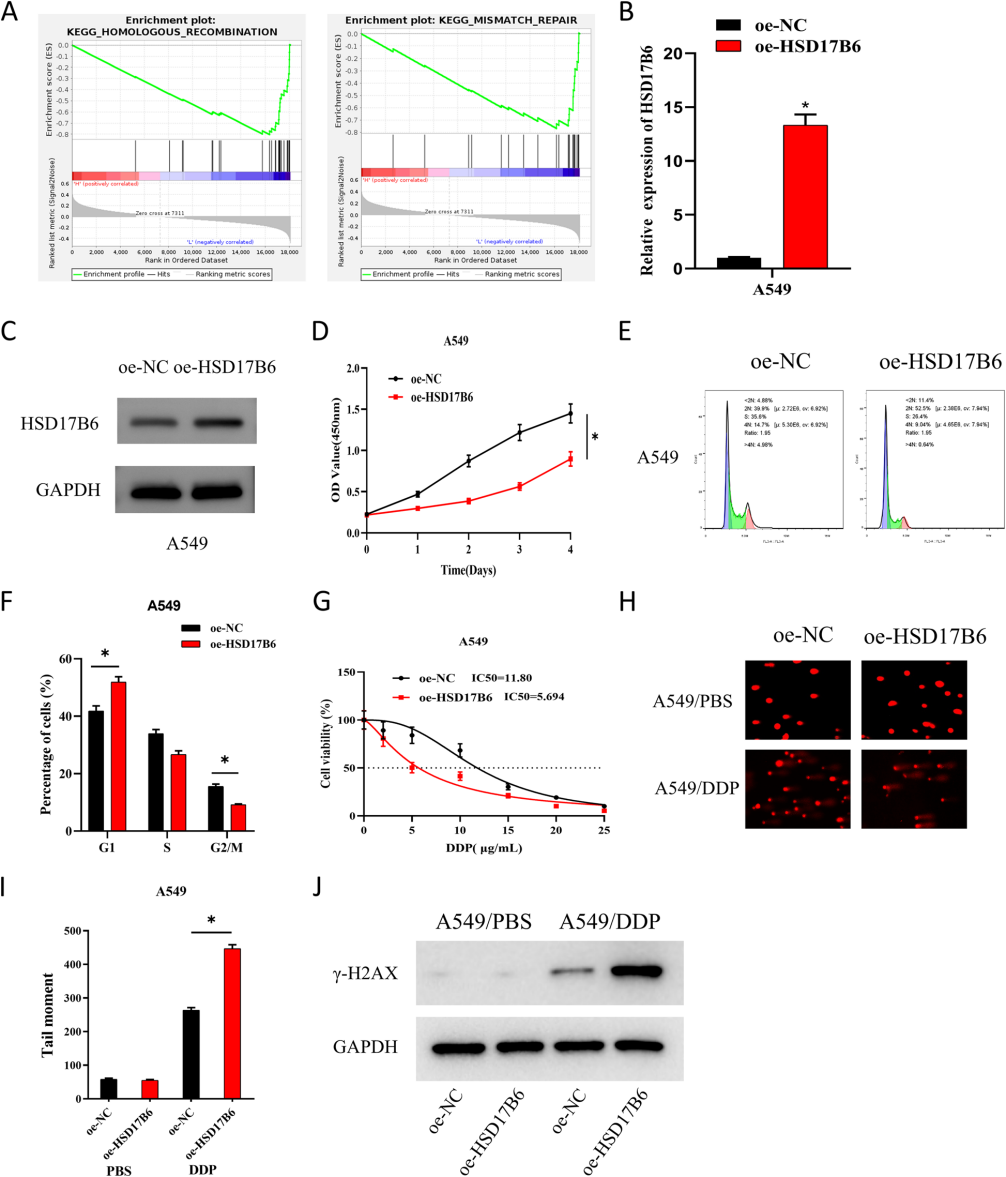
HSD17B6 inhibits LUAD cisplatin resistance via DNA damage. **A** GSEA pathway enrichment analysis of HSD17B6; **B**, **C** qPCR and WB analysis of expression levels of HSD17B6 in different treatment groups. **D** CCK-8 was used to detect the cell viability of different treatment groups. The x-axis represents the incubation days of cells with reagents, and the y-axis represents the OD values. **E**, **F** FCM was used to detect the number of cells in G1, S and G2/M phases. The x-axis represents DNA content, where 2N (DNA content doubles, typically corresponding to G1 and G2 phases) and 4N (DNA content quadruples, typically corresponding to G2 and M phases) indicate different cell cycle stages, and the y-axis represents the count of viable cells; **G** CCK-8 was used to detect the IC_50_ values of the two groups of cells treated with gradient concentrations of cisplatin. **H**, **I**:Comet assay was used to detect DNA damage in the two groups of cells treated with PBS and semi-inhibitory concentration cisplatin. **J** WB was used to detect the expression of DNA damage-related proteins γ-H2AX in cells from different treatment groups. * means *P* < 0.05

To investigate the biological function of HSD17B6, function enrichment analysis of HSD17B6 was performed with GSEA, with results showing that HSD17B6 was mainly enriched in homologous recombination and mismatch repair pathways (Fig. 2A). Based on the above results, we constructed the following cell groups for A549 and H1299: oe-NC and oe-HSD17B6 and assayed the expression of HSD17B6 in different treatment groups by qRT-PCR and WB. The results exhibited that HSD17B6 overexpression considerably raised the expression of HSD17B6 (Fig. 2B, C; Supplementary Fig. 1A-B). As indicated by CCK-8 assay, overexpression of HSD17B6 dramatically inhibited A549 and H1299 cell viability (Fig. 2D; Supplementary Fig. 1C). Overexpression of HSD17B6 significantly inhibited the number of A549 and H1299 cells in the G2/M phase and dramatically raised that in the G1 phase, as measured by FCM for cell cycle (Fig. 2E, F; Supplementary Fig. 1D).

DNA damage has been reported to be linked with chemotherapy resistance [20]. Subsequently, we further explored the impact of HSD17B6 on LUAD cisplatin resistance. We detected the viability of two groups of cells, which were treated with gradient concentrations of cisplatin (DDP) (0, 2, 5, 10, 15, 20, and 25 μg/mL) by CCK-8, and calculated IC_50_ values, finding that HSD17B6 overexpression notably inhibited the IC_50_ values of A549 and H1299 cells (Fig. 2G; Supplementary Fig. 1E). To investigate the association between DNA damage and LUAD cisplatin resistance, we treated both groups of cells with 10 μg/mL cisplatin for 48 h. As measured by comet assay, overexpression of HSD17B6 caused notably greater DNA damage in cisplatin-treated A549 cells than in control group (Fig. 2H, I; Supplementary Fig. 1F). Finally, the expression of DNA damage-related protein γ-H2AX was assayed by WB, with results showing that HSD17B6 overexpression had no prominent difference in the expression of γ-H2AX in PBS-treated A549 and H1299 cells. In contrast, HSD17B6 overexpression significantly promoted the expression of γ-H2AX in cisplatin-treated A549 and H1299 cells (Fig. 2J; Supplementary Fig. 1G). The above results indicated that HSD17B6 could inhibit LUAD cisplatin resistance by DNA damage.

### GATA1 is an upstream transcription factor of HSD17B6

**Fig. 3:**
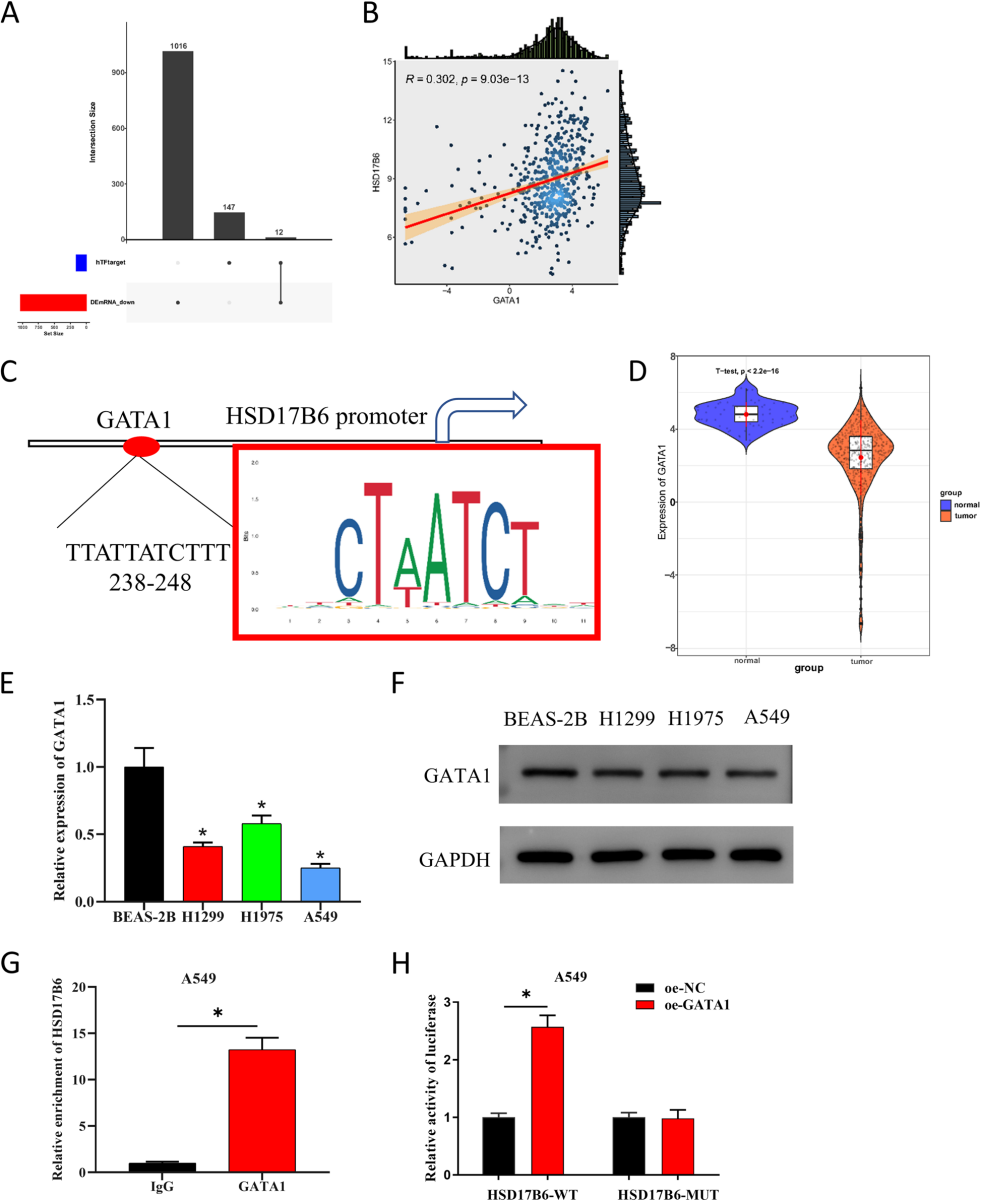
HSD17B6 inhibits LUAD cisplatin resistance via DNA damage. **A** Upset plot was used to visualize the intersection of the upstream potential transcription factors predicted by hTFtarget database and differentially downregulated genes; The x-axis represents the upstream potential transcription factor set of HSD17B6 predicted by hTFtarget, along with the set of differentially downregulated genes analyzed from the TCGA database. The intersection of these sets yields the potential upstream transcription factor set of HSD17B6. The y-axis represents the number of transcription factors and differentially expressed genes. **B** Pearson correlation diagram of GATA1 and HSD17B6; **C** The binding site map of transcription factor GATA1 and HSD17B6 in JASPAR database; The red box indicates multiple binding sites in the first 2000 bp region of the predicted HSD17B6 promoter, and “TTATTATCTTT 238–248” indicates the binding sites of GATA1 and HSD176 with the highest degree of matching with the predicted binding sites; **D** TCGA analysis of expression of GATA1 in LUAD tumor tissues and normal tissues; **E**, **F** qPCR and WB analysis of expression of GATA1 in human LUAD cell lines and human bronchial epithelial cells; **G** ChIP showed that GATA1 bound to the predicted site in the 2000 bp region upstream of the HSD17B6 promoter; **H** Dual-luciferase reporter assay further verified the interaction between GATA1 and HSD17B6 promoter. * means *P* < 0.05

To investigate the potential transcriptional regulators of HSD17B6, the upstream potential transcription factors were predicted by hTFtarget (Supplementary Table 1) and intersected with the differential down-regulated genes to obtain 12 potential transcription factors (Fig. 3A; Supplementary Fig. 2A). As Pearson correlation analysis revealed, GATA1 had prominent positive correlation with HSD17B6 (Fig. 3B; Supplementary Fig. 2B). Besides, JASPAR prediction identified a binding site in the first 2000 bp region of HSD17B6 promoter (Fig. 3C). GATA1 has been reported lowly expressed in LUAD tissues [21], and GATA1 can function as a transcription activator [22]. Subsequently, we found that the gene was down-regulated in LUAD tissues by t-test analysis (Fig. 3D). The expression of GATA1 in human LUAD cell lines H1299, H1975 and A549 was prominently lower than that in human bronchial epithelial cells BEAS-2B (Fig. 3E, F). ChIP assay subsequently showed that HSD17B was significantly enriched by GATA1-specific antibodies, as compared with negative control IgG antibodies (Fig. 3G). As dual-luciferase reporter assay revealed, overexpression of GATA1 considerably raised the luciferase activity of A549 cells (Fig. 3H). Thus, we ascertained the presence of a targeted relationship between GATA1 and HSD17B. These results suggested that HSD17B6 had an upstream transcription factor GATA1 and that GATA1 was down-regulated in LUAD.

### GATA1 activates HSD17B6 to inhibit LUAD cisplatin resistance via DNA damage

**Fig. 4:**
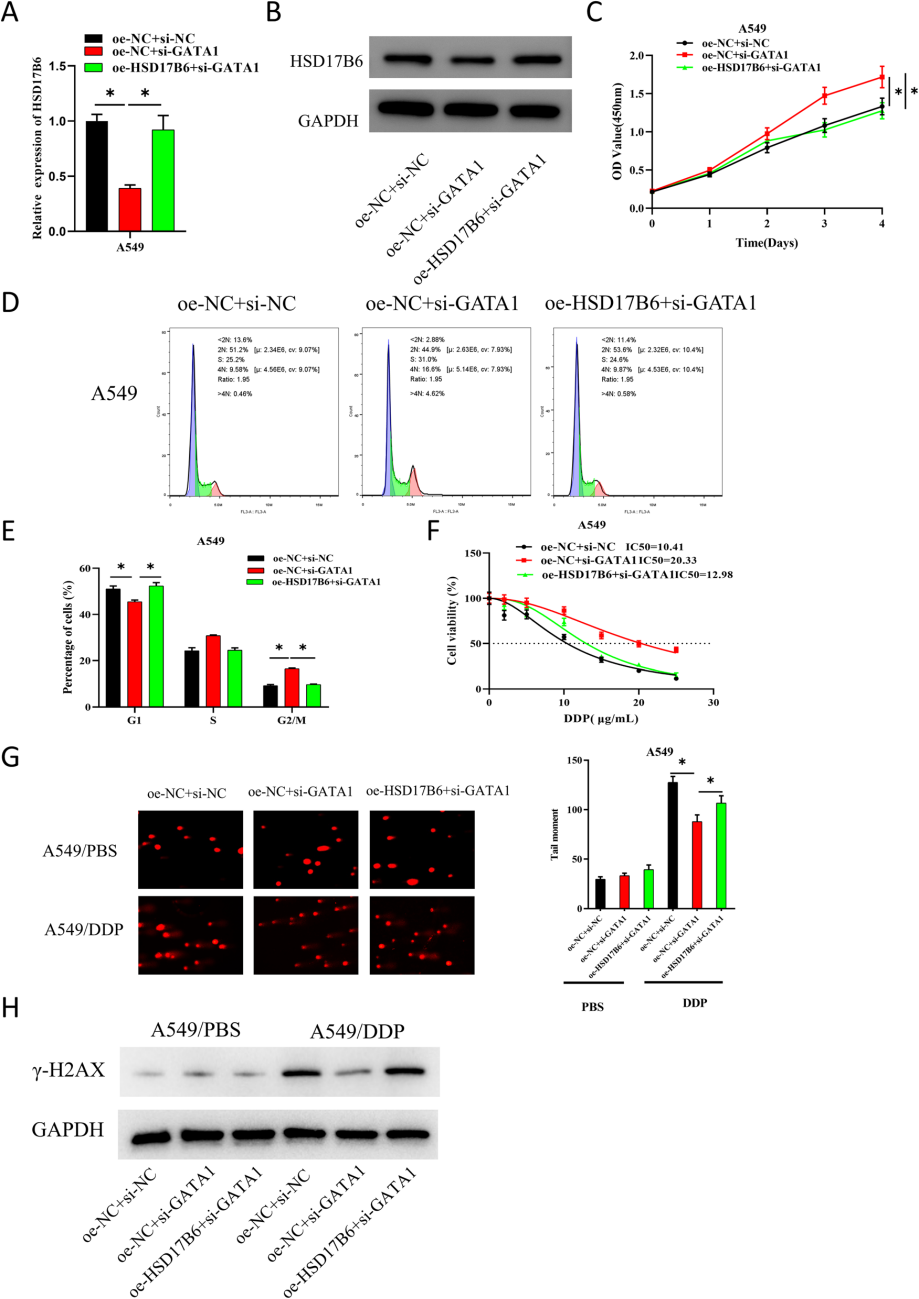
GATA1 inhibits LUAD cisplatin resistance by activating HSD17B6 via DNA damage. **A**, **B** qRT-PCR and WB was used to detect the expression of HSD17B6 in A549 cells in each treatment group; **C** CCK-8 was used to detect the viability of A549 cells in each treatment group; **D**, **E** FCM was used to detect the number of cells in G1, S and G2/M phases in each treatment group; **F** CCK-8 was used to detect the IC_50_ value of each treatment group treated with gradient concentrations of cisplatin; **G** Comet assay was used to detect DNA damage in each group of cells treated with PBS and semi-inhibitory concentration of cisplatin; **H** WB was used to detect the expression of DNA damage-related proteins in cells of each treatment group. * means *P* < 0.05

In the above study, we mentioned that HSD17B6 inhibited LUAD cisplatin resistance via DNA damage, and GATA1 was an upstream regulator of HSD17B6. To investigate the effect of GATA1 on LUAD by activating HSD17B6, we constructed the following cell groups based on A549: oe-NC + si-NC, oe-NC + si-GATA1, and oe-HSD17B6 + si-GATA1. Based on results of qRT-PCR and WB, knockdown of GATA1 could significantly down-regulate the expression of HSD17B6, while overexpression of HSD17B6 significantly reversed the inhibitory effect of low GATA1 expression on HSD17B6 expression (*P* < 0.05) (Fig. 4A, B). Cell viability of each group was assayed by CCK-8, with outcomes showing that knockdown of GATA1 could considerably enhance A549 cell viability compared with the control, while overexpression of HSD17B6 considerably offset the promoting effect of low GATA1 expression on A549 cell viability (Fig. 4C). Cell cycle was assayed by FCM, which displayed that knockdown of GATA1 significantly raised the number of A549 cells in G2/M phase and shortened the number of cells in G1 phase, while overexpression of HSD17B6 significantly reversed the promoting effect of low expression of GATA1 on cell cycle progression (*P* < 0.05) (Fig. 4D, E). Subsequently, we explored the impact of GATA1 on LUAD cisplatin resistance. CCK-8 was utilized to assay the viability of two groups of cells treated with gradient concentrations of cisplatin (0, 2, 5, 10, 15, 20 and 25 μg/mL), and IC_50_ values were calculated. The IC_50_ value of GATA1 knockdown cells was found to be significantly higher than that of control group. In contrast, overexpression of HSD17B6 significantly reversed the promoting effect on IC_50_ value by low GATA1 expression (*P* < 0.05) (Fig. 4F). To investigate the link between DNA damage and LUAD cisplatin resistance, cells in each group had cisplatin treatments at a semi-inhibitory concentration (10 μg/mL) for 48 h. As measured by comet assay, knockdown of GATA1 resulted in an observably lower level of DNA damage in cisplatin-treated A549 cells. On the contrary, overexpression of HSD17B6 dramatically offset the inhibitory effect of GATA1 knockdown on DNA damage (Fig. 4G). Finally, the expression of DNA damage-related protein γ-H2AX was detected by WB. As outcomes established, knockdown of GATA1 dramatically reduced the expression of γ-H2AX in A549 cells treated with cisplatin. However, overexpression of HSD17B6 largely offset the inhibitory effect of low GATA1 expression on DNA damage-related proteins (Fig. 4H). The above results suggested that GATA1 activated HSD17B6 to inhibit LUAD cisplatin resistance via DNA damage.

## Discussion

Lung cancer is the deadliest type of cancer, and LUAD is most common subtype in lung cancer [23]. Cisplatin, one of the commonly used chemotherapeutic drugs, which has vast application in curing various solid tumors, such as ovarian cancer [24], cervical cancer [25] and lung cancer [26]. In cancer, many patients possess intrinsic resistance or chemoresistance to cisplatin, posing a primary challenge to cisplatin-based anticancer therapy [27]. The mechanism of tumor resistance to cisplatin mainly includes the accumulation of drugs in cells, the inactivation of drug solutes, and the DNA damage response that changes with the enhancement of DNA repair process. Among these mechanisms, enhanced DNA damage repair is a driving force of cisplatin resistance [28]. For instance, Fang et al. [29] ascertained that DUSP1 enhanced cisplatin resistance in gallbladder cancer by activating p38 pathway and DNA damage repair system. Xu et al*.* [30] reported that down-regulated MARK2 inhibits cisplatin resistance of osteosarcoma stem cells by modulating DNA damage repair. Here, HSD17B6 was found lowly expressed in LUAD and related to DNA damage repair. Cell experiments showed that HSD17B6 could inhibit LUAD cisplatin resistance through DNA damage. Therefore, HSD17B6 may be a feasible target for curing chemoresistance in LUAD.

In the process of exploring specific mechanism of HSD17B6 affecting LUAD cisplatin resistance, HSD17B6 was found to had an upstream transcription factor GATA1, which was ascertained to be a transcriptional activator of HSD17B6 by molecular experiments. GATA1 is the original member of GATA transcription factor protein family, which possesses two zinc finger domains which are highly conserved, namely N-terminal finger and C-terminal finger [31]. GATA1 has been ascertained to be associated with cell phenotypes and development of solid tumors such as colorectal cancer [32], breast cancer [33] and ovarian cancer [22]. As Shi et al*.* [34] revealed, GATA1 is up-regulated in cholangiocarcinoma, and knockout of GATA1 gene hampers malignant behavior of cholangiocarcinoma cells via PI3K/AKT pathway disruption. Additionally, GATA1 is related to tumor chemotherapy resistance. Li et al*.* [32] ascertained that GATA1-induced LINC01503 upregulation enhances carboplatin resistance in ovarian cancer by upregulating PD-L1 through sponging miR-766-5p. Chang et al. [31] reported that GATA1 facilitates gemcitabine resistance in pancreatic cancer via an anti-apoptotic pathway. Strikingly, GATA1 expression was significantly downregulated in LUAD in our study, and silencing GATA1 could inhibit DNA damage, thereby promoting cisplatin resistance in LUAD. In addition, overexpressing HSD17B6 on this basis can restore the inhibitory effect on DNA damage in LUAD cells caused by knocking out GATA1. Due to the presence of tumor heterogeneity, Sangiorgio et al. [35] ascertained that GATA1 expression was down-regulated in the prefibrotic and fibrotic stages of primary myelofibrosis, as well as in the myelofibrosis in other myeloproliferative tumors. Here, we discovered that GATA1 activated HSD17B6 to inhibit LUAD cisplatin resistance via DNA damage, which further clarified the molecular mechanism of LUAD chemotherapy resistance.

In summary, our study demonstrated the interaction between GATA1 and HSD17B6 and its role in LUAD cisplatin resistance. We dug out that the transcription factor GATA1 activated the expression of HSD17B6, which in turn inhibited cisplatin resistance in LUAD by promoting DNA damage, breeding new insights into the therapeutic target for LUAD chemotherapy resistance. However, our study still has certain limitations. For instance, the authenticity of this theory could not be validated through clinical or animal experiments, and the specific molecular regulatory mechanisms between GATA1 and HSD17B6 were not explored. Therefore, further experiments need to be designed in the future for verification. Taken together, our outcome demonstrated the influence of GATA1/HSD17B6 regulatory axis in cisplatin resistance in LUAD, adding weight to the importance of therapeutic targets to enhancing tumor chemotherapy sensitivity.

## Supplementary Information

Supplementary Material 1.

Supplementary Material 2.

Supplementary Material 3: Supplementary Fig. 1. HSD17B6 inhibits LUAD cisplatin resistance via DNA damage. A-B: qPCR and WB analysis of expression levels of HSD17B6 in different treatment groups. C: CCK-8 was used to detect the cell viability of different treatment groups. D: FCM was used to detect the number of cells in G1, S and G2/M phases. E: CCK-8 was used to detect the IC_50_ values of the two groups of cells treated with gradient concentrations of cisplatin. F: Comet assay was used to detect DNA damage in the two groups of cells treated with PBS and semi-inhibitory concentration cisplatin. G: WB was used to detect the expression of DNA damage-related proteins γ-H2AX in cells from different treatment groups. * means *P*<0.05.

Supplementary Material 4: Supplementary Fig. 2. Potential transcription factors upstream of HSD17B6. A: Venn diagram of potential upstream transcription factors intersecting with differentially down-regulated genes. B: Pearson correlation analysis of HSD17B6 with 12 potential transcription factors.

Supplementary Material 5: Supplementary Table 1. 147 candidate transcription factors predicted by hTFtarget.

## Data Availability

The data and materials in the current study are available from the corresponding author on reasonable request.
