# Supplementary figures and images for: GATA1 activates HSD17B6 to improve efficiency of cisplatin in lung adenocarcinoma via DNA damage
Source: Genes Environ. 2024 Dec 18;46:27. doi: 10.1186/s41021-024-00321-9 (PMC11654308; doi:10.1186/s41021-024-00321-9)

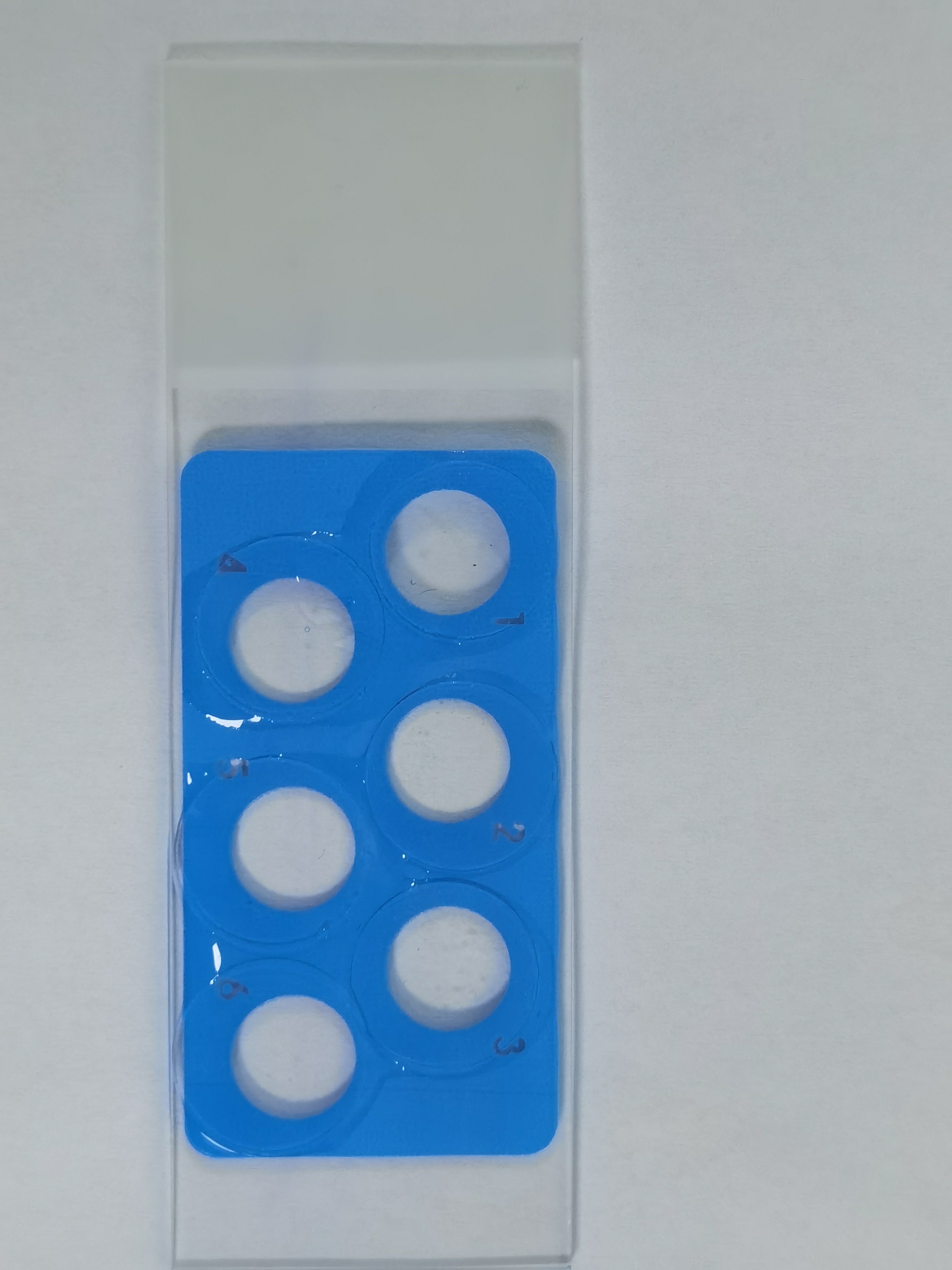

Supplement: Supplementary file 1 — Supplementary Material 1. [file 41021_2024_321_MOESM1_ESM.jpg]

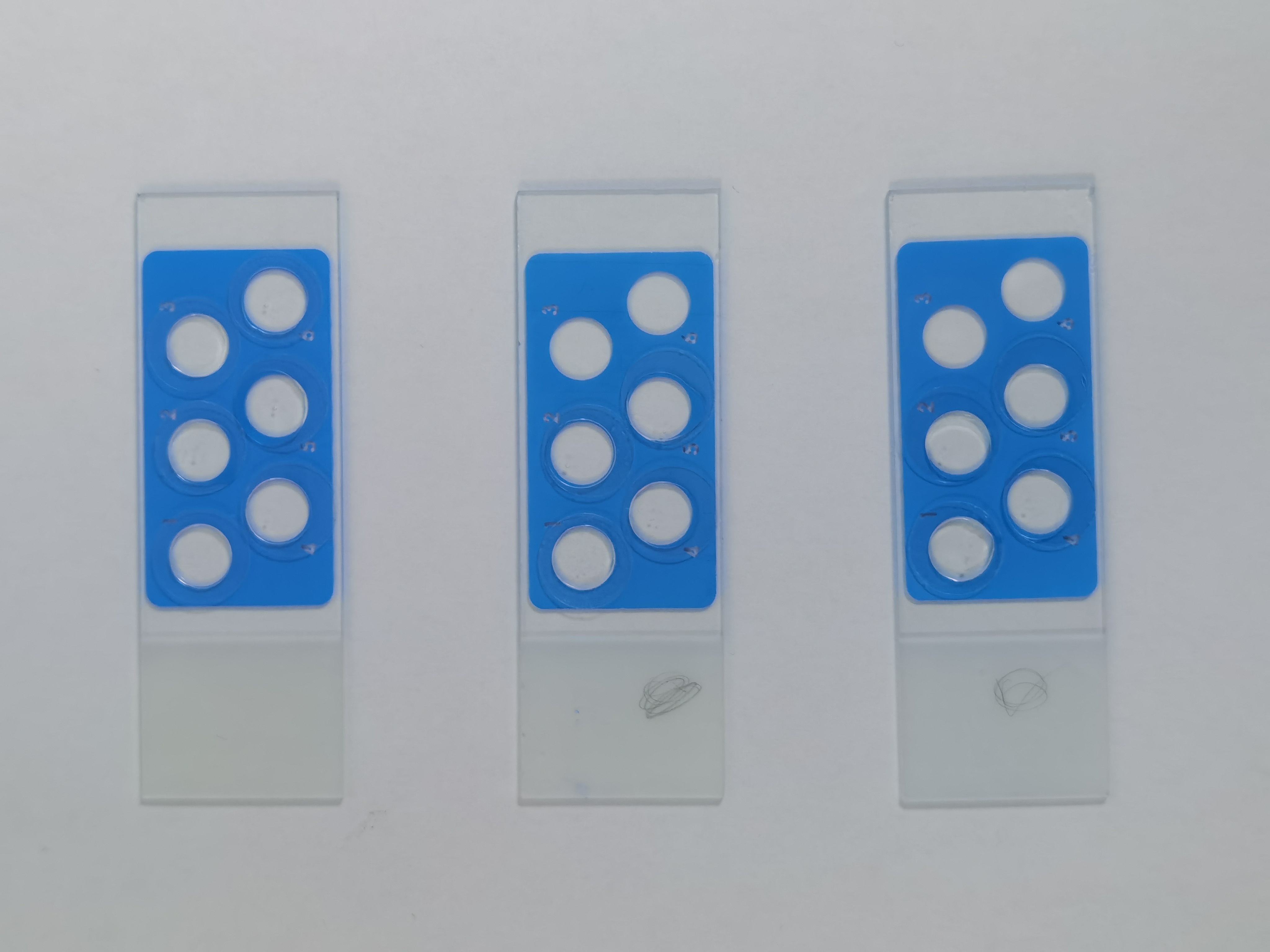

Supplement: Supplementary file 2 — Supplementary Material 2. [file 41021_2024_321_MOESM2_ESM.jpg]

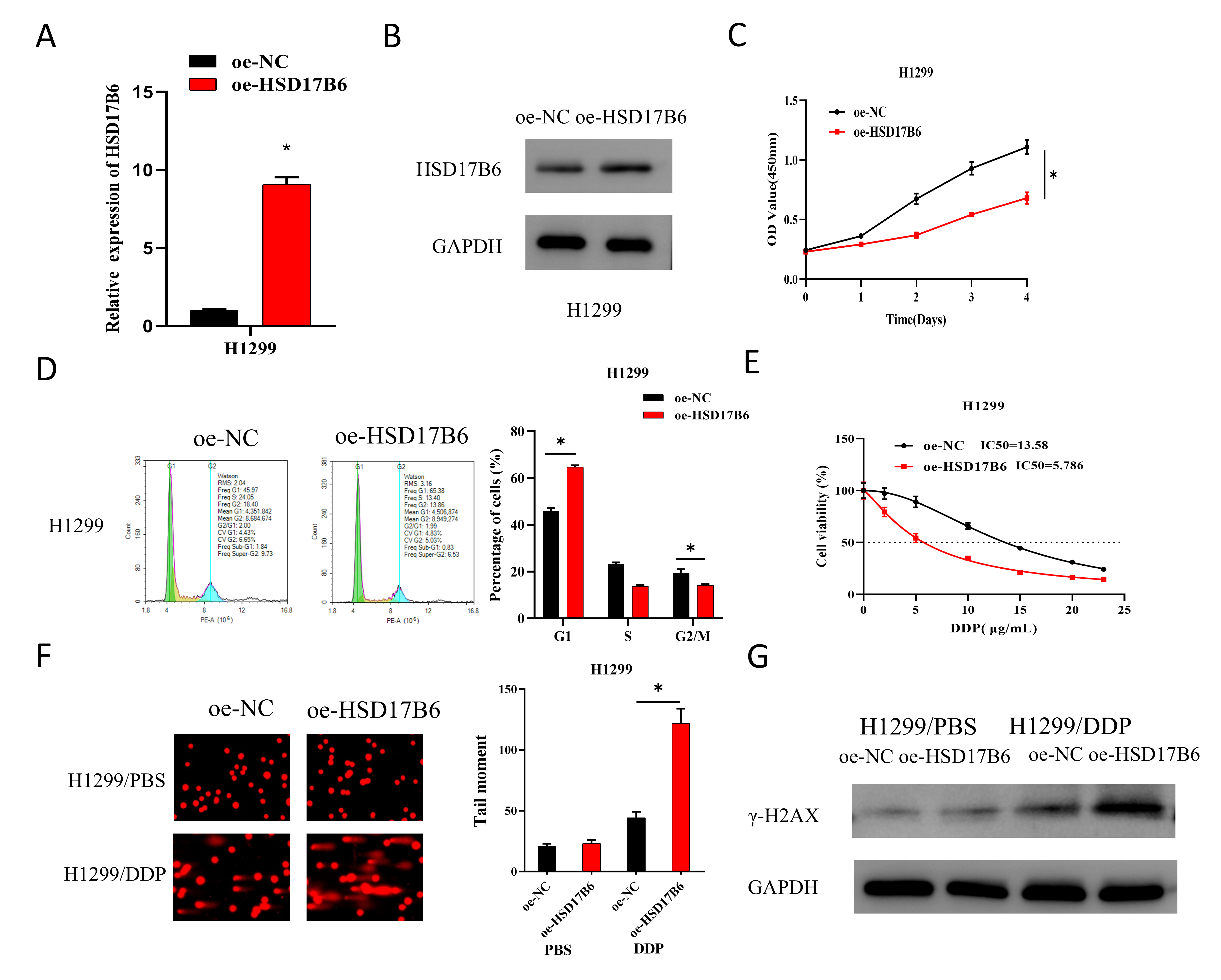

Supplement: Supplementary file 3 — Supplementary Material 3: Supplementary Fig. 1. HSD17B6 inhibits LUAD cisplatin resistance via DNA damage. A-B: qPCR and WB analysis of expression levels of HSD17B6 in different treatment groups. C: CCK-8 was used to detect the cell viability of different treatment groups. D: FCM was used to detect the number of cells in G1, S and G2/M phases. E: CCK-8 was used to detect the IC50 values of the two groups of cells treated with gradient concentrations of cisplatin. F: Comet assay was used to detect DNA damage in the two groups of cells treated with PBS and semi-inhibitory concentration cisplatin. G: WB was used to detect the expression of DNA damage-related proteins γ-H2AX in cells from different treatment groups. * means P<0.05. [file 41021_2024_321_MOESM3_ESM.tif]

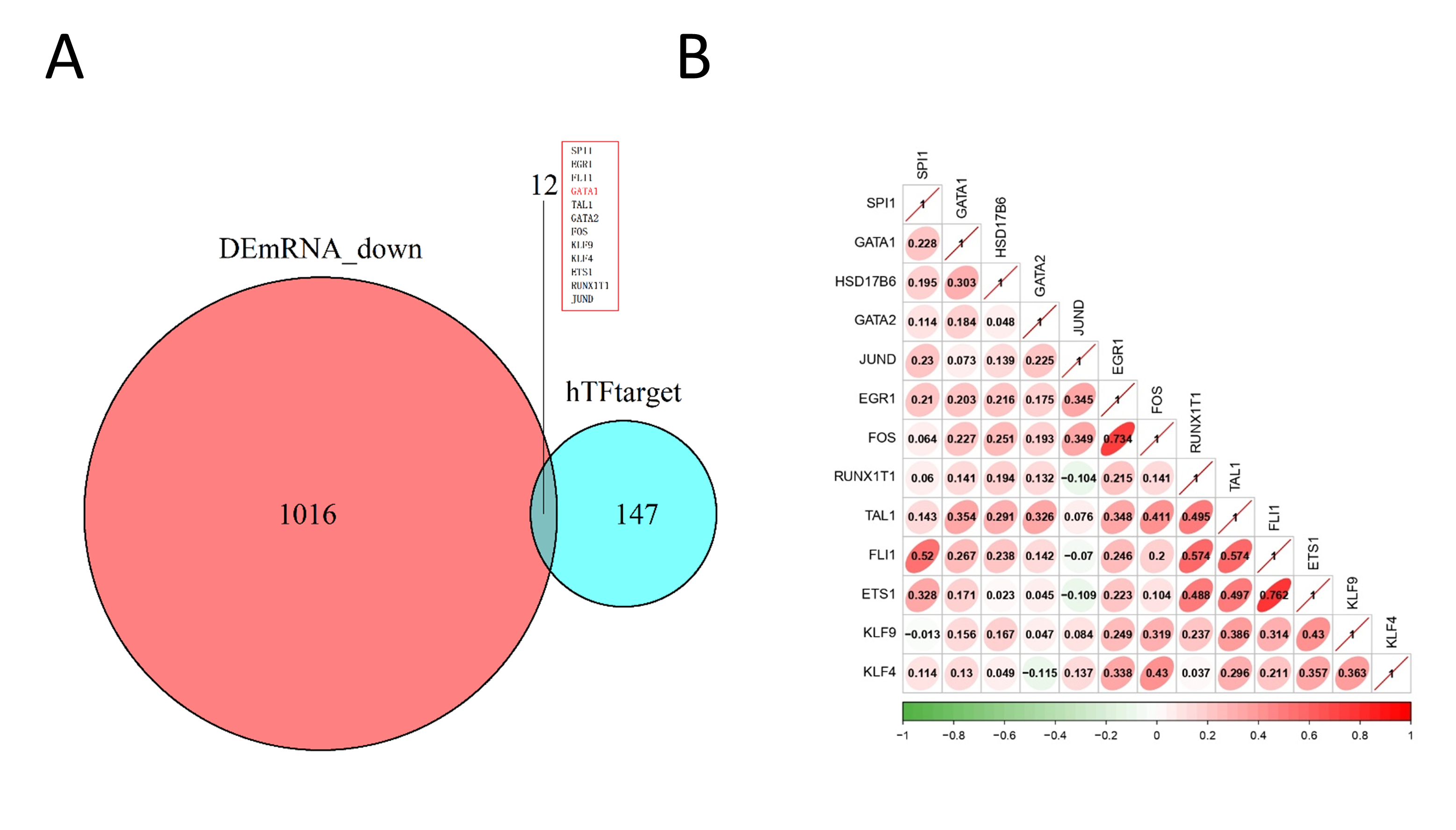

Supplement: Supplementary file 4 — Supplementary Material 4: Supplementary Fig. 2. Potential transcription factors upstream of HSD17B6. A: Venn diagram of potential upstream transcription factors intersecting with differentially down-regulated genes. B: Pearson correlation analysis of HSD17B6 with 12 potential transcription factors. [file 41021_2024_321_MOESM4_ESM.tif]
